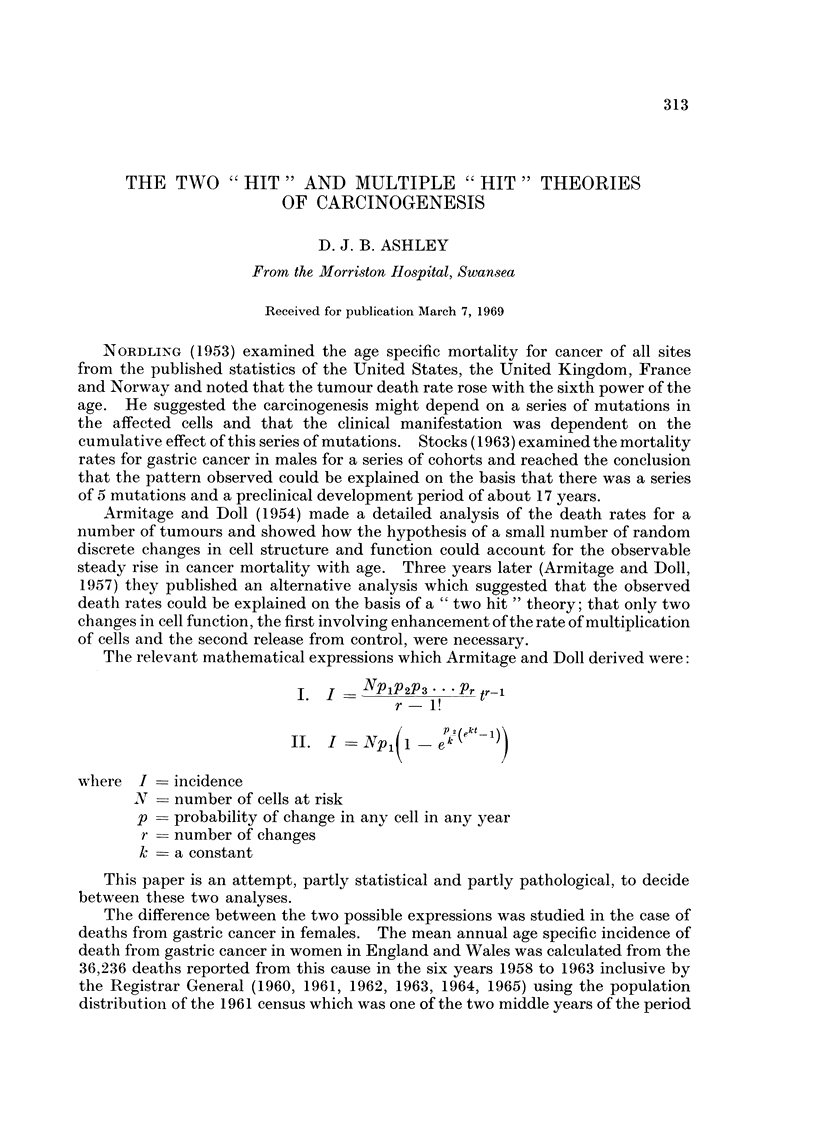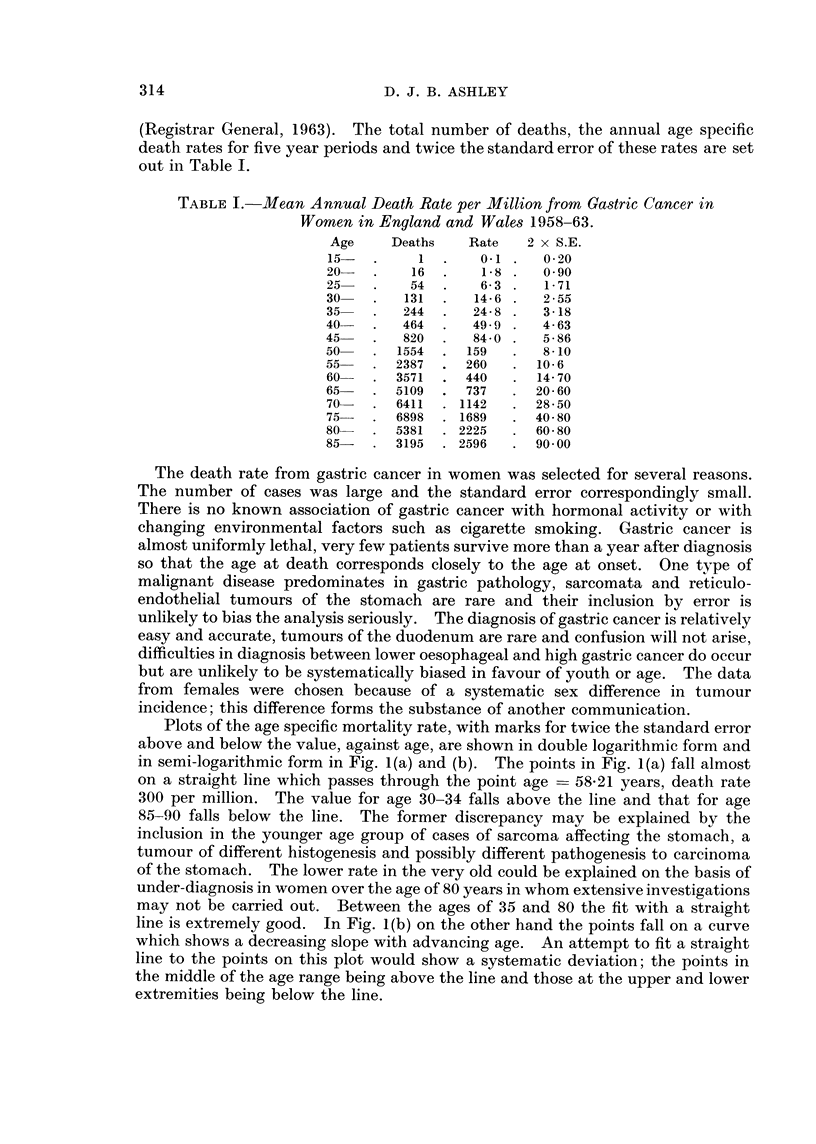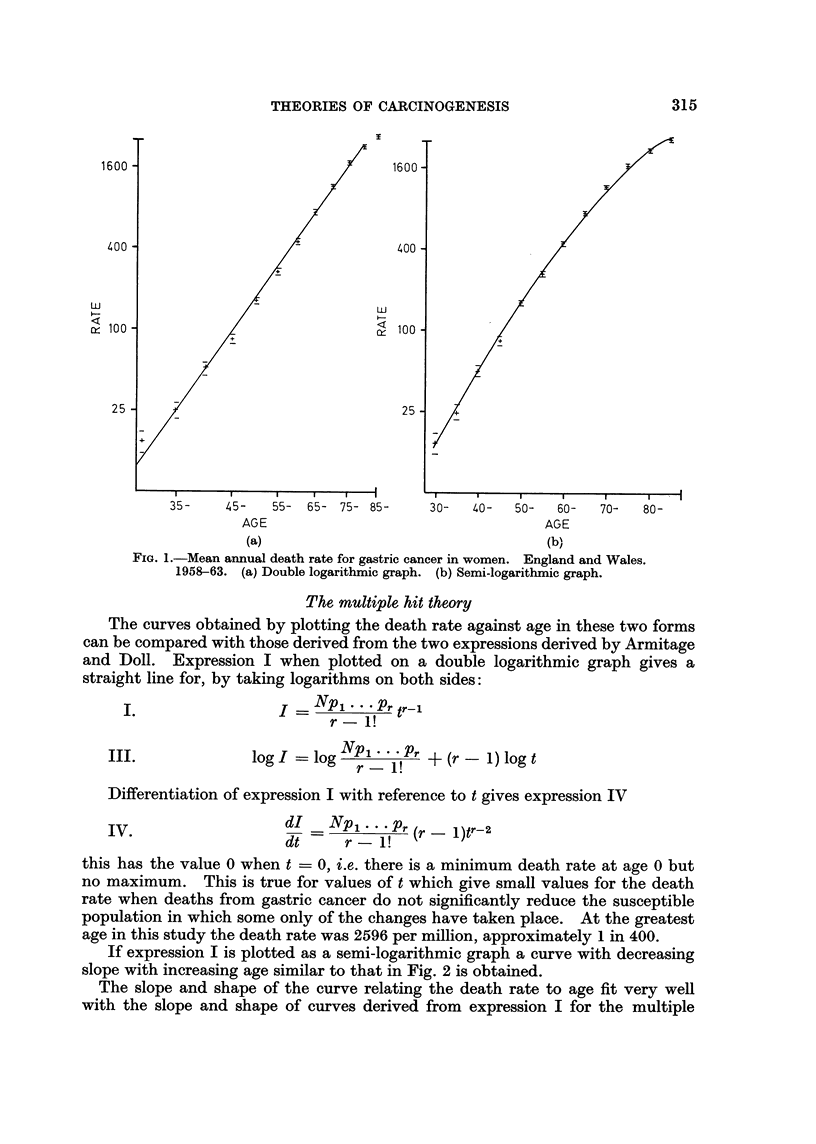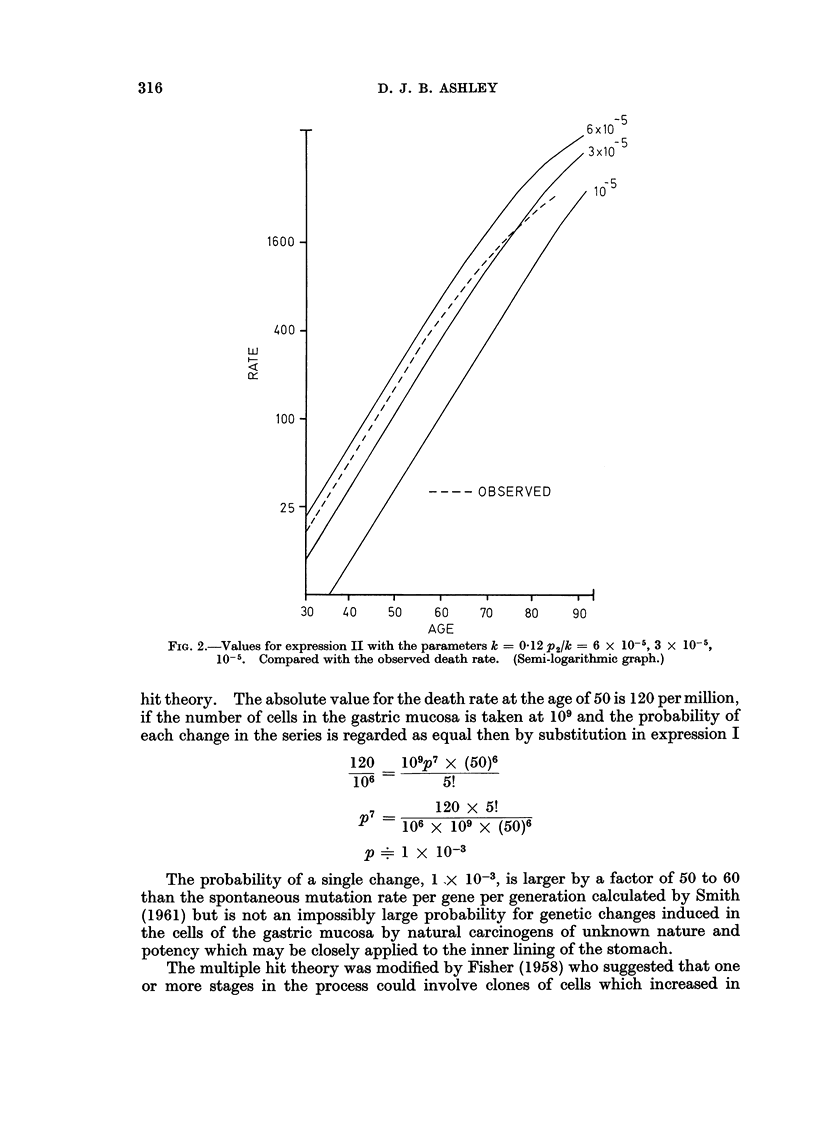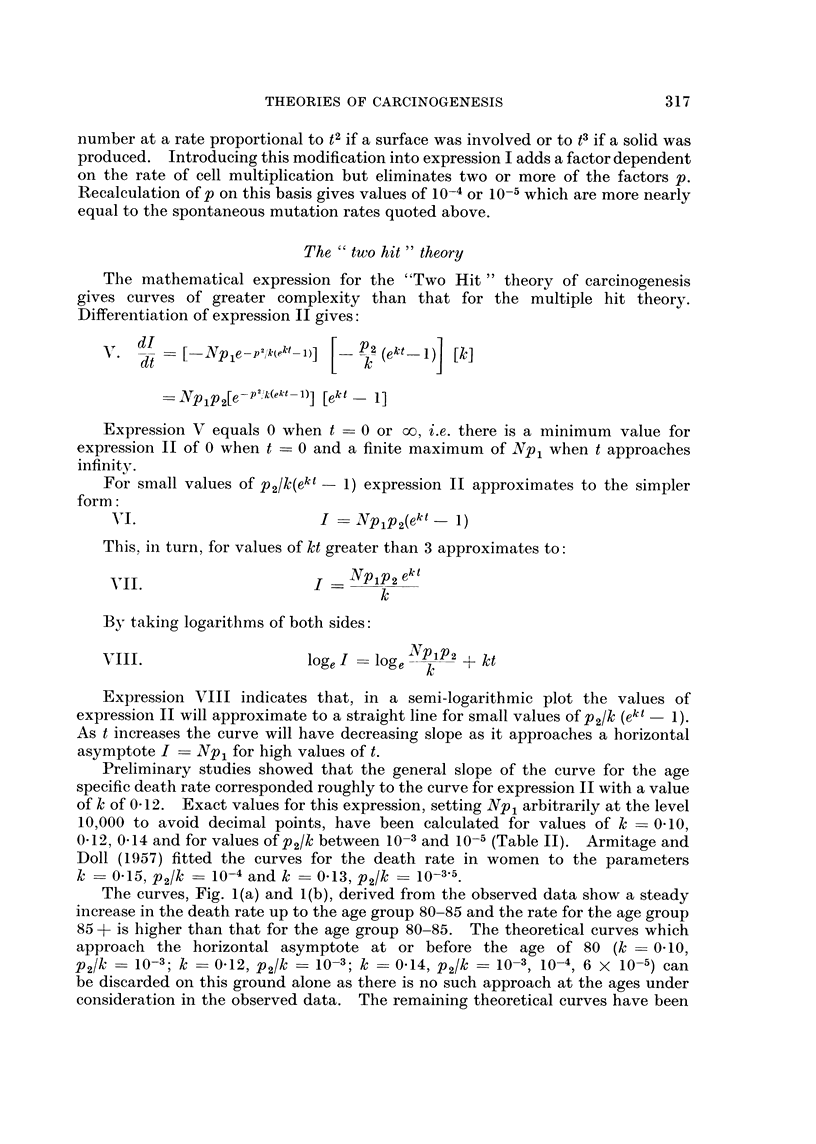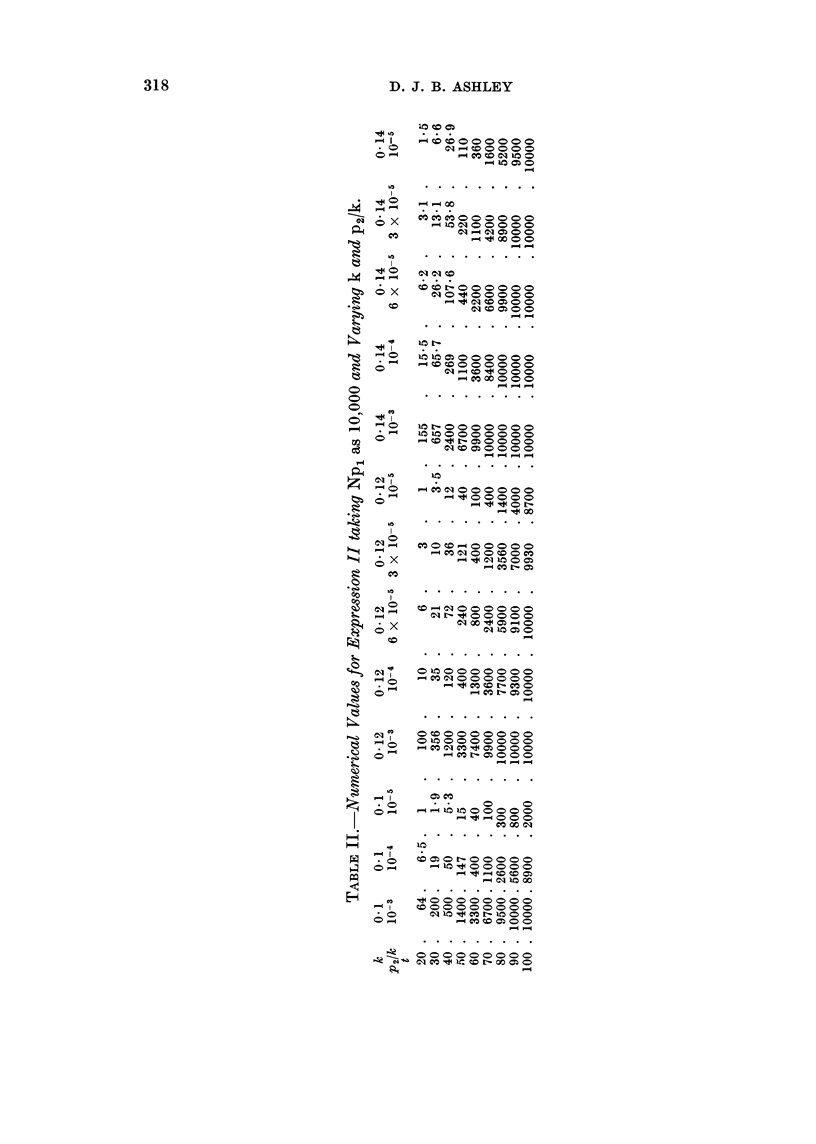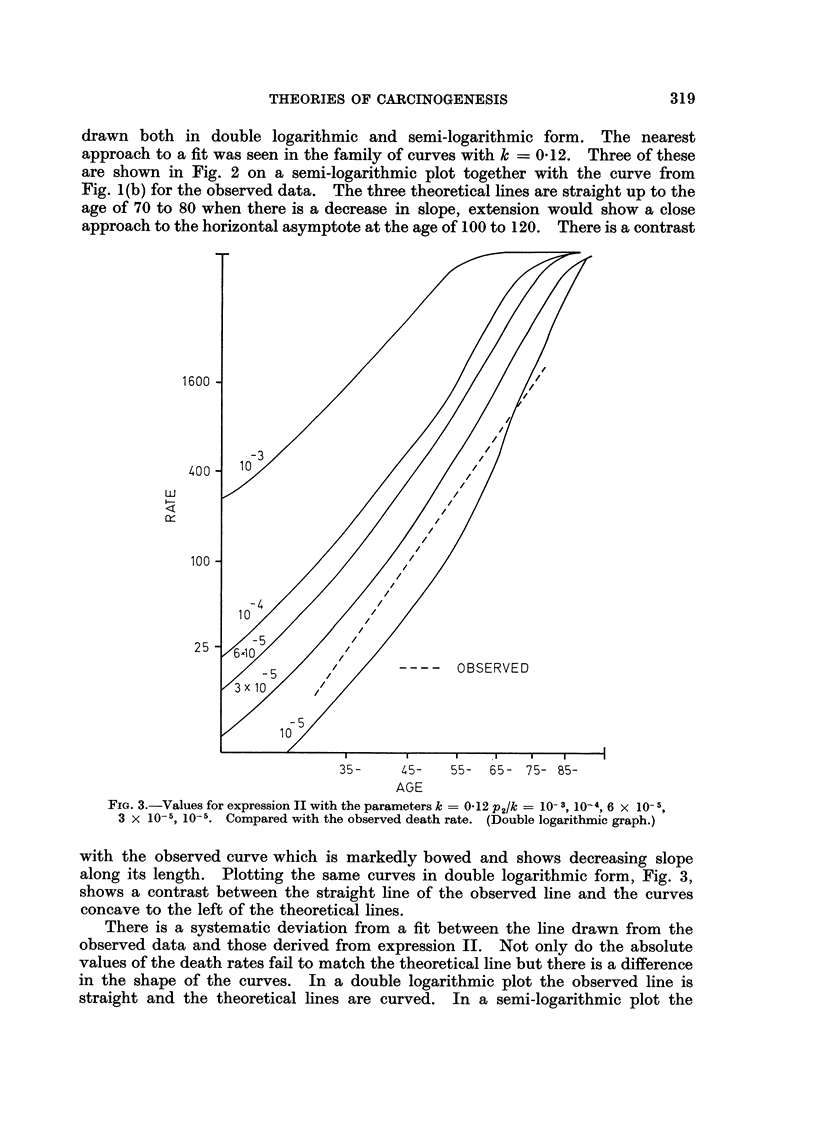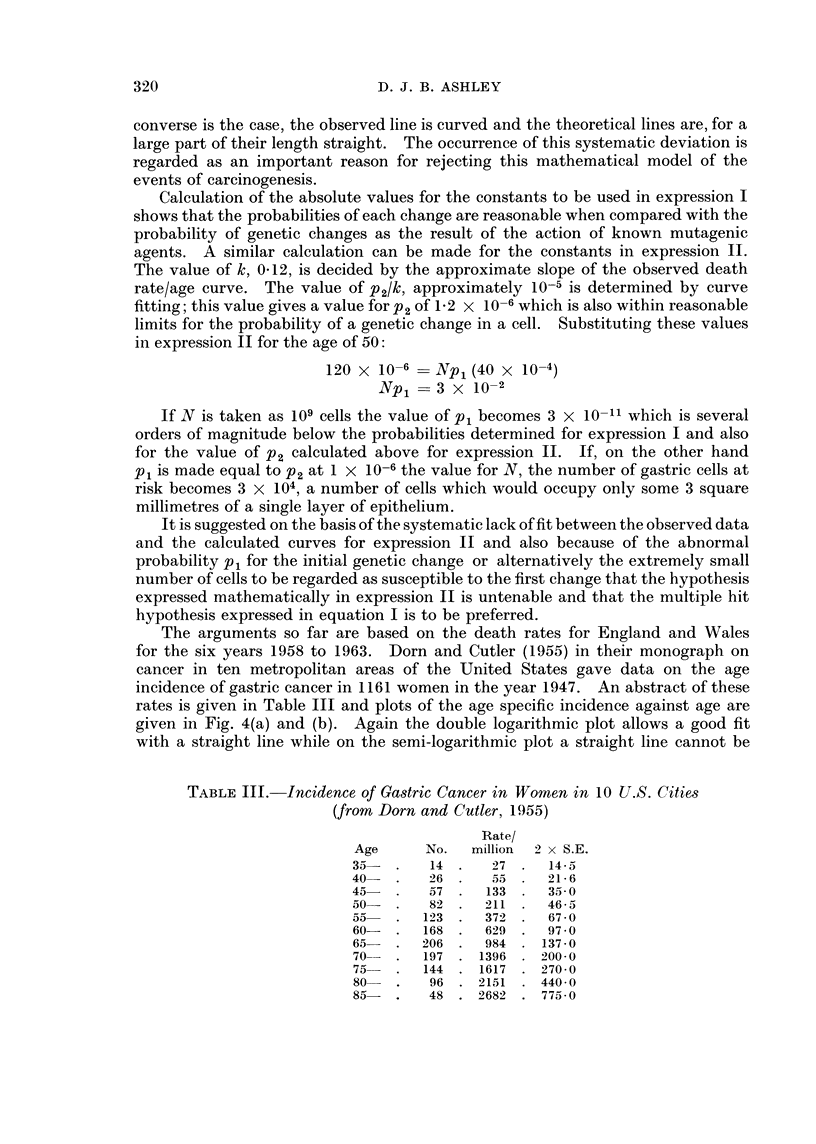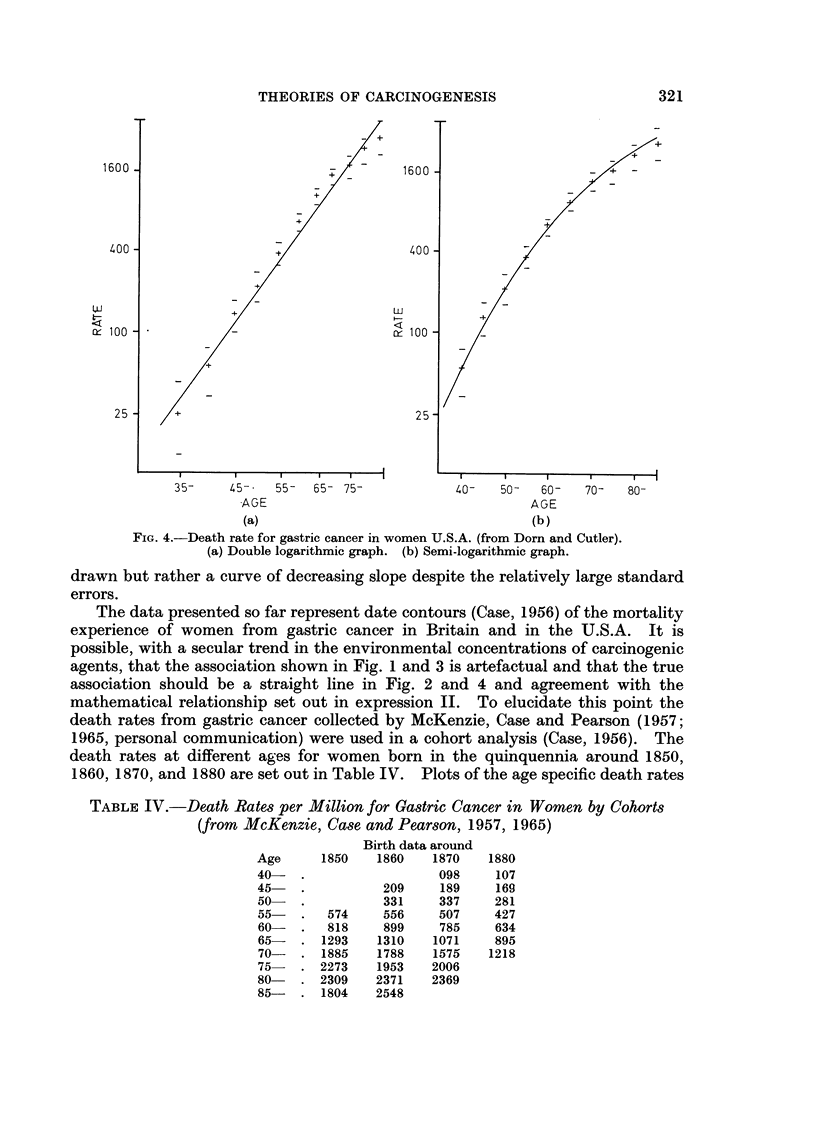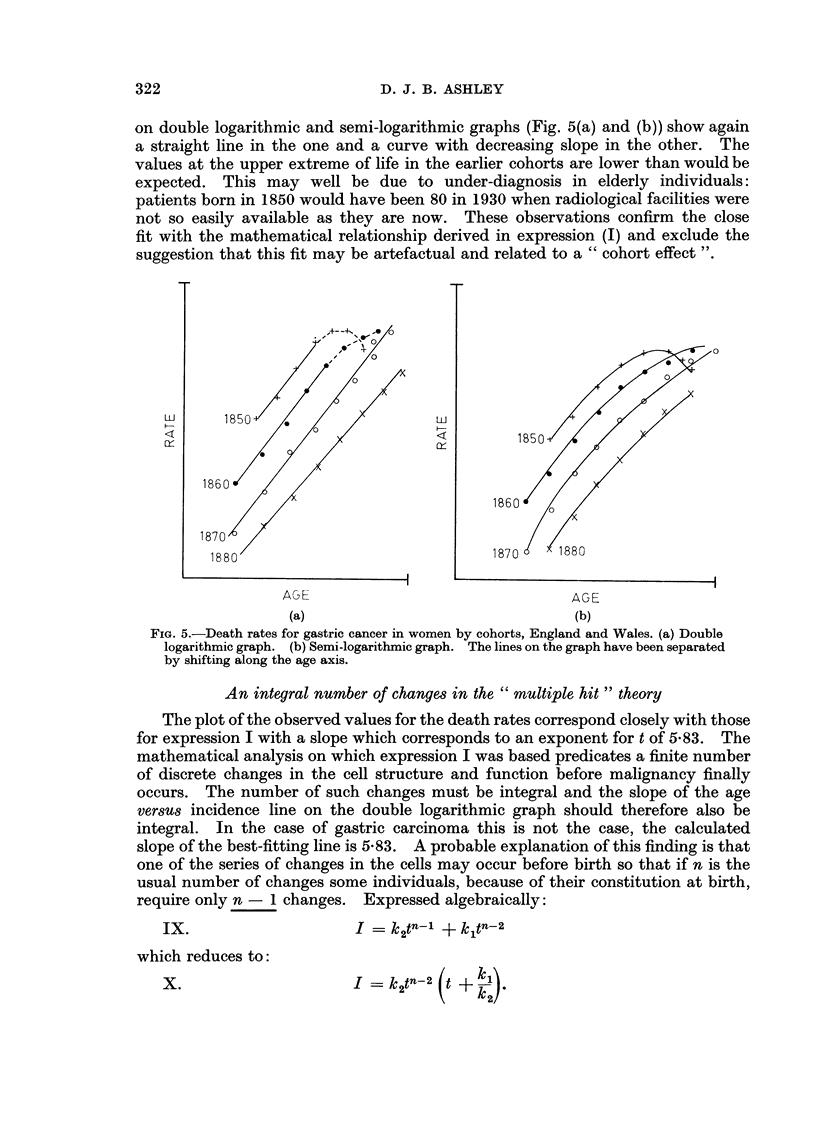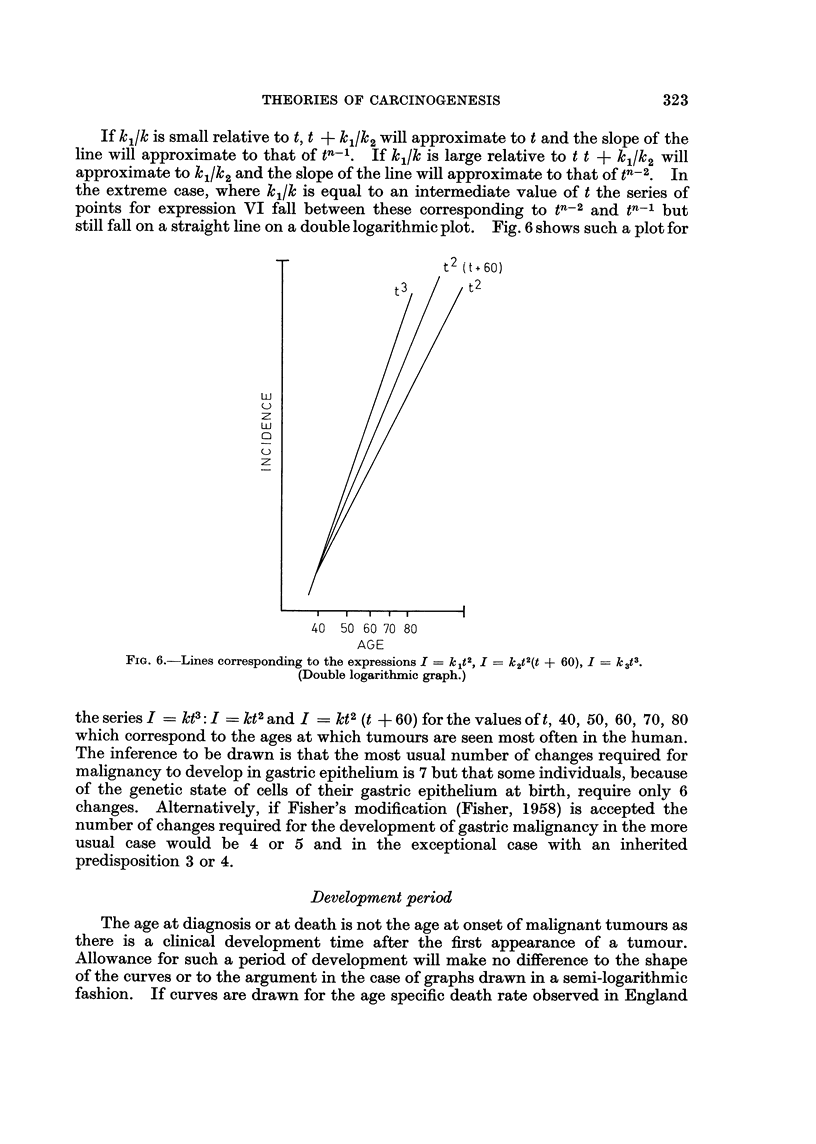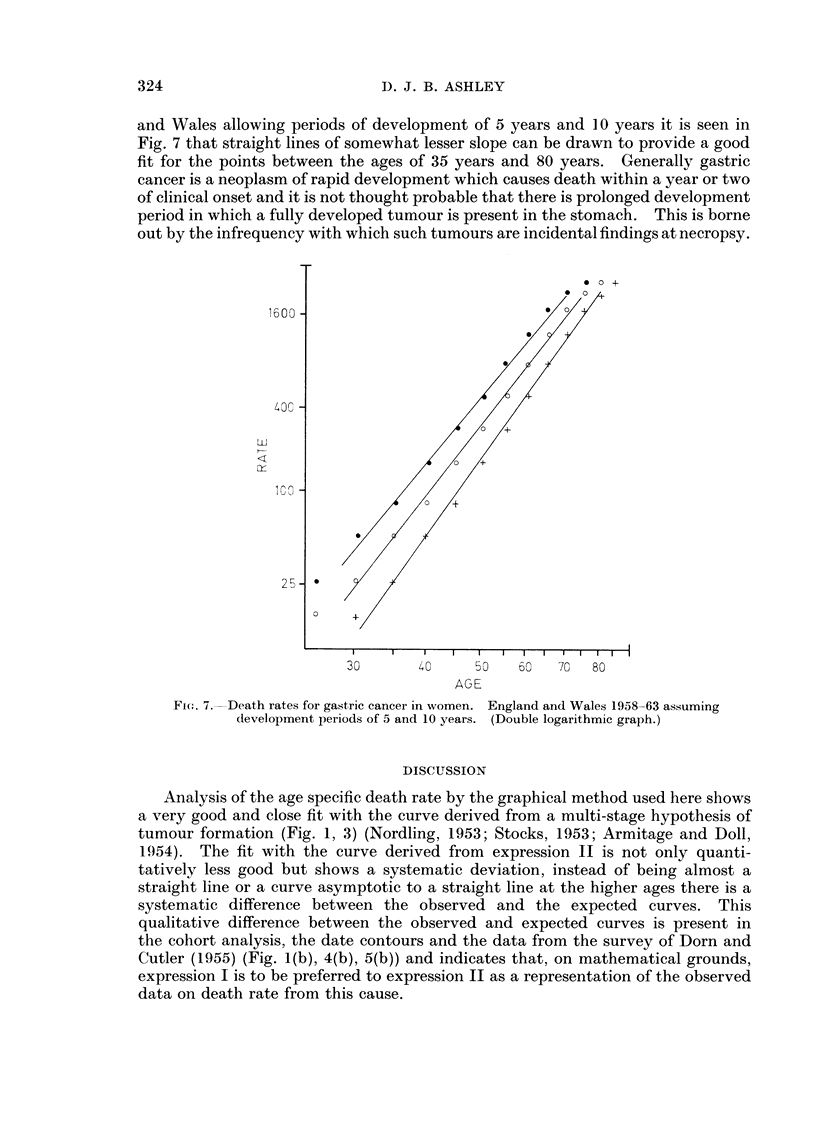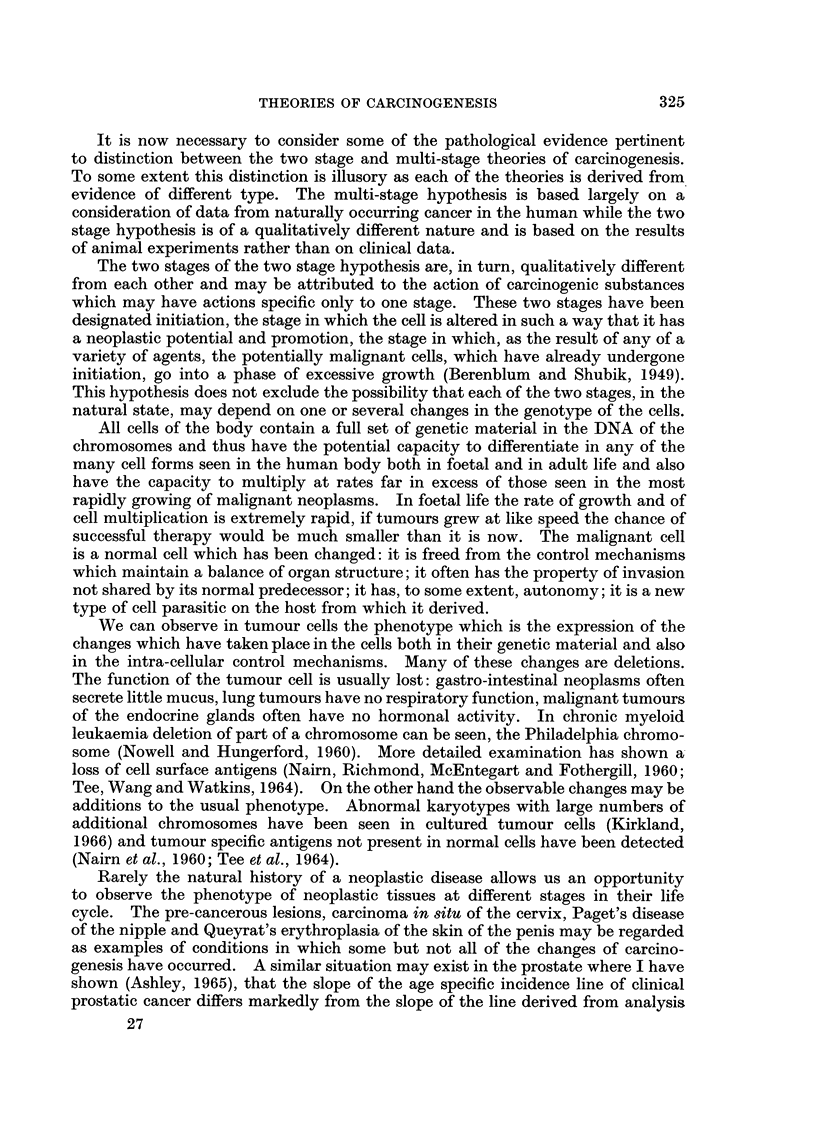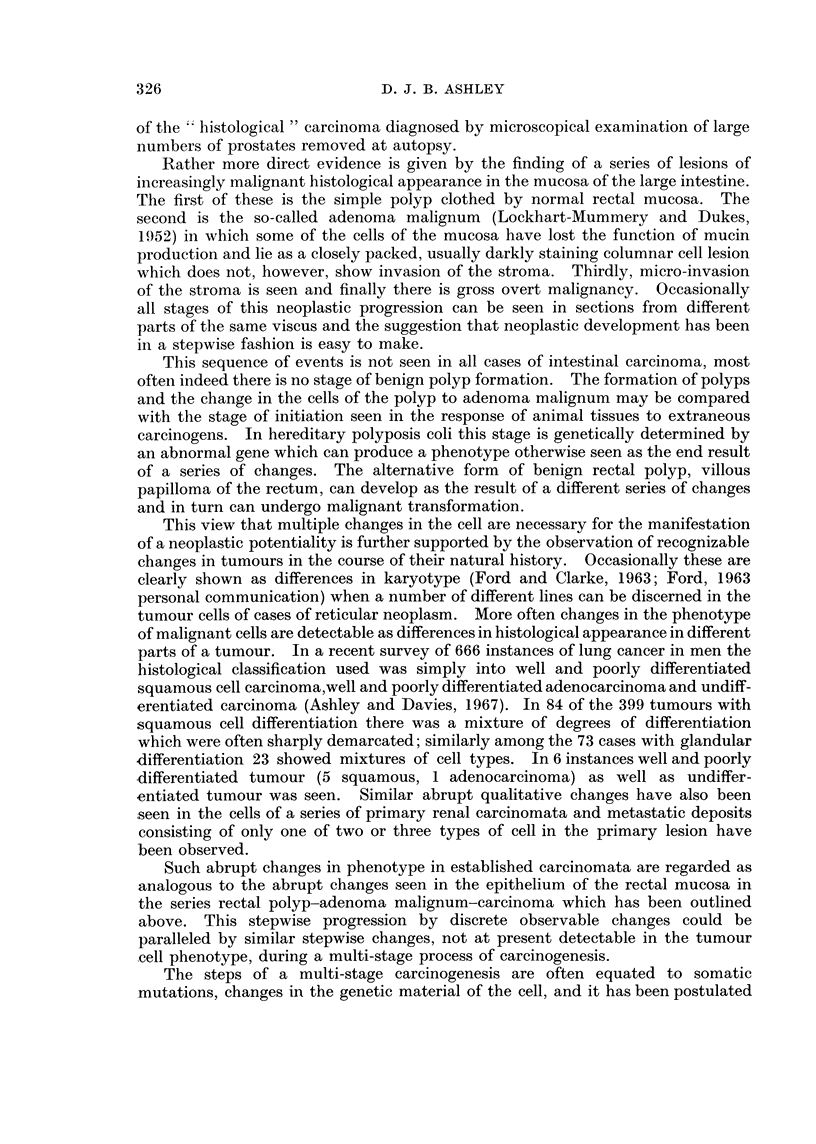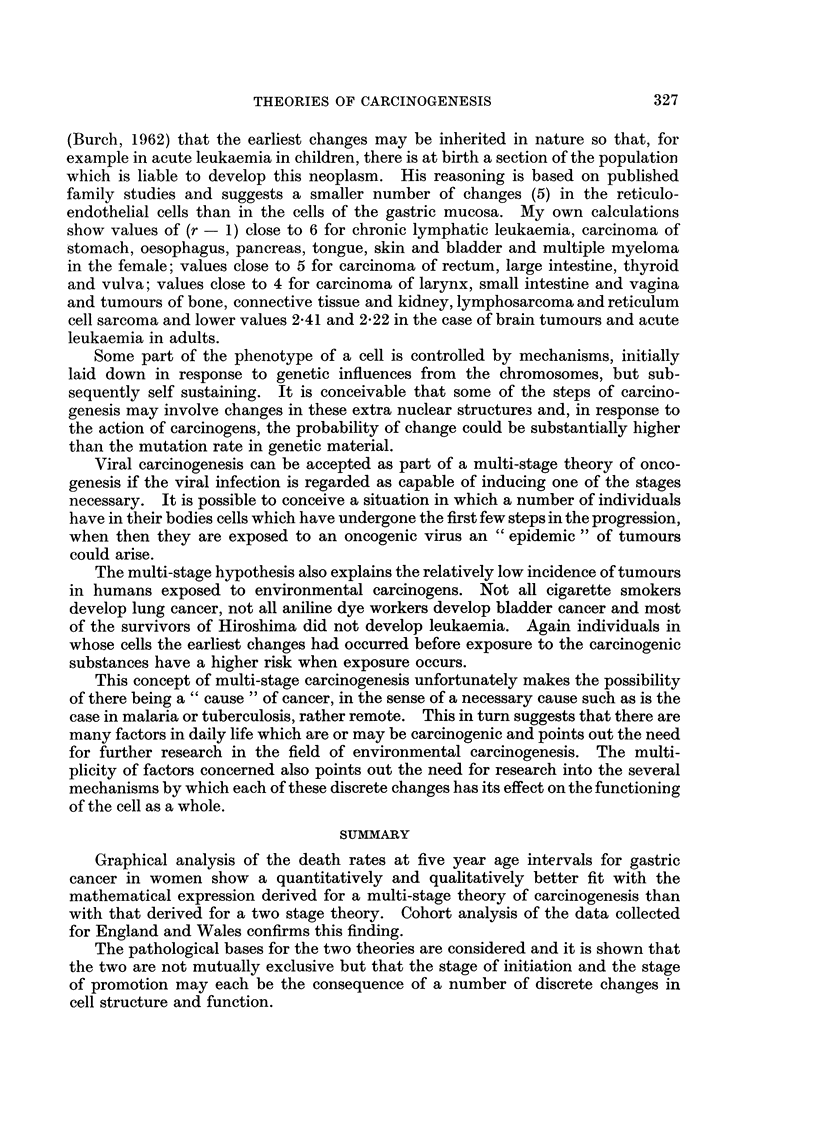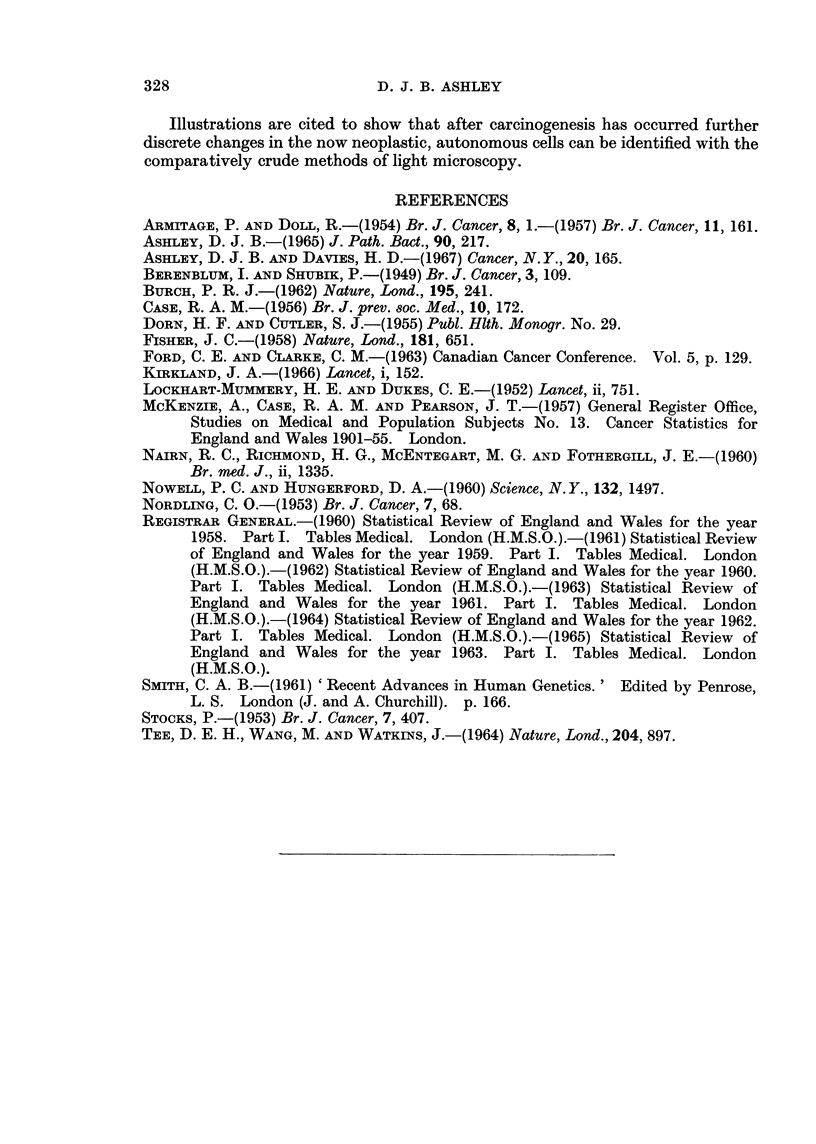# The two "hit" and multiple "hit" theories of carcinogenesis.

**DOI:** 10.1038/bjc.1969.41

**Published:** 1969-06

**Authors:** D. J. Ashley


### THE TWO "HIT" AND MULTIPLE "HIT" THEORIES OF CARCINOGENESIS

D. J. B. ASHLEY

*From the Morriston Hospital, Swansea*

Received for publication March 7, 1969

NORDLING (1953) examined the age specific mortality for cancer of all sites from the published statistics of the United States, the United Kingdom, France and Norway and noted that the tumour death rate rose with the sixth power of the age. He suggested the carcinogenesis might depend on a series of mutations in the affected cells and that the clinical manifestation was dependent on the cumulative effect of this series of mutations. Stocks (1963) examined the mortality rates for gastric cancer in males for a series of cohorts and reached the conclusion that the pattern observed could be explained on the basis that there was a series of 5 mutations and a preclinical development period of about 17 years.

Armitage and Doll (1954) made a detailed analysis of the death rates for a number of tumours and showed how the hypothesis of a small number of random discrete changes in cell structure and function could account for the observable steady rise in cancer mortality with age. Three years later (Armitage and Doll, 1957) they published an alternative analysis which suggested that the observed death rates could be explained on the basis of a "two hit" theory; that only two changes in cell function, the first involving enhancement of the rate of multiplication of cells and the second release from control, were necessary.

The relevant mathematical expressions which Armitage and Doll derived were:

$$\text{I.} \quad I = \frac{Np_1p_2p_3 \ldots p_r}{r - 1!} t^{r-1}$$

$$\text{II.} \quad I = Np_1\left(1 - e^{\frac{p_2}{k}(e^{kt}-1)}\right)$$

where $I$ = incidence
$N$ = number of cells at risk
$p$ = probability of change in any cell in any year
$r$ = number of changes
$k$ = a constant

This paper is an attempt, partly statistical and partly pathological, to decide between these two analyses.

The difference between the two possible expressions was studied in the case of deaths from gastric cancer in females. The mean annual age specific incidence of death from gastric cancer in women in England and Wales was calculated from the 36,236 deaths reported from this cause in the six years 1958 to 1963 inclusive by the Registrar General (1960, 1961, 1962, 1963, 1964, 1965) using the population distribution of the 1961 census which was one of the two middle years of the period

(Registrar General, 1963). The total number of deaths, the annual age specific death rates for five year periods and twice the standard error of these rates are set out in Table I.

TABLE I.—*Mean Annual Death Rate per Million from Gastric Cancer in Women in England and Wales* 1958–63.

| Age | Deaths | Rate | 2 × S.E. |
|---|---|---|---|
| 15— | 1 | 0·1 | 0·20 |
| 20— | 16 | 1·8 | 0·90 |
| 25— | 54 | 6·3 | 1·71 |
| 30— | 131 | 14·6 | 2·55 |
| 35— | 244 | 24·8 | 3·18 |
| 40— | 464 | 49·9 | 4·63 |
| 45— | 820 | 84·0 | 5·86 |
| 50— | 1554 | 159 | 8·10 |
| 55— | 2387 | 260 | 10·6 |
| 60— | 3571 | 440 | 14·70 |
| 65— | 5109 | 737 | 20·60 |
| 70— | 6411 | 1142 | 28·50 |
| 75— | 6898 | 1689 | 40·80 |
| 80— | 5381 | 2225 | 60·80 |
| 85— | 3195 | 2596 | 90·00 |

The death rate from gastric cancer in women was selected for several reasons. The number of cases was large and the standard error correspondingly small. There is no known association of gastric cancer with hormonal activity or with changing environmental factors such as cigarette smoking. Gastric cancer is almost uniformly lethal, very few patients survive more than a year after diagnosis so that the age at death corresponds closely to the age at onset. One type of malignant disease predominates in gastric pathology, sarcomata and reticulo-endothelial tumours of the stomach are rare and their inclusion by error is unlikely to bias the analysis seriously. The diagnosis of gastric cancer is relatively easy and accurate, tumours of the duodenum are rare and confusion will not arise, difficulties in diagnosis between lower oesophageal and high gastric cancer do occur but are unlikely to be systematically biased in favour of youth or age. The data from females were chosen because of a systematic sex difference in tumour incidence; this difference forms the substance of another communication.

Plots of the age specific mortality rate, with marks for twice the standard error above and below the value, against age, are shown in double logarithmic form and in semi-logarithmic form in Fig. 1(a) and (b). The points in Fig. 1(a) fall almost on a straight line which passes through the point age = 58·21 years, death rate 300 per million. The value for age 30–34 falls above the line and that for age 85–90 falls below the line. The former discrepancy may be explained by the inclusion in the younger age group of cases of sarcoma affecting the stomach, a tumour of different histogenesis and possibly different pathogenesis to carcinoma of the stomach. The lower rate in the very old could be explained on the basis of under-diagnosis in women over the age of 80 years in whom extensive investigations may not be carried out. Between the ages of 35 and 80 the fit with a straight line is extremely good. In Fig. 1(b) on the other hand the points fall on a curve which shows a decreasing slope with advancing age. An attempt to fit a straight line to the points on this plot would show a systematic deviation; the points in the middle of the age range being above the line and those at the upper and lower extremities being below the line.

FIG. 1.—Mean annual death rate for gastric cancer in women. England and Wales. 1958–63. (a) Double logarithmic graph. (b) Semi-logarithmic graph.

*The multiple hit theory*

The curves obtained by plotting the death rate against age in these two forms can be compared with those derived from the two expressions derived by Armitage and Doll. Expression I when plotted on a double logarithmic graph gives a straight line for, by taking logarithms on both sides:

I. $$I = \frac{Np_1 \ldots p_r}{r - 1!} t^{r-1}$$

III. $$\log I = \log \frac{Np_1 \ldots p_r}{r - 1!} + (r - 1) \log t$$

Differentiation of expression I with reference to $t$ gives expression IV

IV. $$\frac{dI}{dt} = \frac{Np_1 \ldots p_r}{r - 1!} (r - 1) t^{r-2}$$

this has the value 0 when $t = 0$, *i.e.* there is a minimum death rate at age 0 but no maximum. This is true for values of $t$ which give small values for the death rate when deaths from gastric cancer do not significantly reduce the susceptible population in which some only of the changes have taken place. At the greatest age in this study the death rate was 2596 per million, approximately 1 in 400.

If expression I is plotted as a semi-logarithmic graph a curve with decreasing slope with increasing age similar to that in Fig. 2 is obtained.

The slope and shape of the curve relating the death rate to age fit very well with the slope and shape of curves derived from expression I for the multiple

FIG. 2.—Values for expression II with the parameters $k = 0{\cdot}12\, p_2/k = 6 \times 10^{-5}$, $3 \times 10^{-5}$, $10^{-5}$. Compared with the observed death rate. (Semi-logarithmic graph.)

hit theory. The absolute value for the death rate at the age of 50 is 120 per million, if the number of cells in the gastric mucosa is taken at $10^9$ and the probability of each change in the series is regarded as equal then by substitution in expression I

$$\frac{120}{10^6} = \frac{10^9 p^7 \times (50)^6}{5!}$$

$$p^7 = \frac{120 \times 5!}{10^6 \times 10^9 \times (50)^6}$$

$$p \doteqdot 1 \times 10^{-3}$$

The probability of a single change, $1 \times 10^{-3}$, is larger by a factor of 50 to 60 than the spontaneous mutation rate per gene per generation calculated by Smith (1961) but is not an impossibly large probability for genetic changes induced in the cells of the gastric mucosa by natural carcinogens of unknown nature and potency which may be closely applied to the inner lining of the stomach.

The multiple hit theory was modified by Fisher (1958) who suggested that one or more stages in the process could involve clones of cells which increased in number at a rate proportional to $t^2$ if a surface was involved or to $t^3$ if a solid was produced. Introducing this modification into expression I adds a factor dependent on the rate of cell multiplication but eliminates two or more of the factors $p$. Recalculation of $p$ on this basis gives values of $10^{-4}$ or $10^{-5}$ which are more nearly equal to the spontaneous mutation rates quoted above.

### *The " two hit " theory*

The mathematical expression for the "Two Hit " theory of carcinogenesis gives curves of greater complexity than that for the multiple hit theory. Differentiation of expression II gives:

$$\text{V.} \quad \frac{dI}{dt} = [-Np_1 e^{-p^2/k(e^{kt}-1)}] \left[-\frac{p_2}{k}(e^{kt}-1)\right] [k]$$

$$= Np_1 p_2 [e^{-p^2/k(e^{kt}-1)}] [e^{kt} - 1]$$

Expression V equals 0 when $t = 0$ or $\infty$, *i.e.* there is a minimum value for expression II of 0 when $t = 0$ and a finite maximum of $Np_1$ when $t$ approaches infinity.

For small values of $p_2/k(e^{kt} - 1)$ expression II approximates to the simpler form:

$$\text{VI.} \qquad I = Np_1 p_2 (e^{kt} - 1)$$

This, in turn, for values of $kt$ greater than 3 approximates to:

$$\text{VII.} \qquad I = \frac{Np_1 p_2 e^{kt}}{k}$$

By taking logarithms of both sides:

$$\text{VIII.} \qquad \log_e I = \log_e \frac{Np_1 p_2}{k} + kt$$

Expression VIII indicates that, in a semi-logarithmic plot the values of expression II will approximate to a straight line for small values of $p_2/k\ (e^{kt} - 1)$. As $t$ increases the curve will have decreasing slope as it approaches a horizontal asymptote $I = Np_1$ for high values of $t$.

Preliminary studies showed that the general slope of the curve for the age specific death rate corresponded roughly to the curve for expression II with a value of $k$ of 0·12. Exact values for this expression, setting $Np_1$ arbitrarily at the level 10,000 to avoid decimal points, have been calculated for values of $k = 0{\cdot}10$, 0·12, 0·14 and for values of $p_2/k$ between $10^{-3}$ and $10^{-5}$ (Table II). Armitage and Doll (1957) fitted the curves for the death rate in women to the parameters $k = 0{\cdot}15$, $p_2/k = 10^{-4}$ and $k = 0{\cdot}13$, $p_2/k = 10^{-3{\cdot}5}$.

The curves, Fig. 1(a) and 1(b), derived from the observed data show a steady increase in the death rate up to the age group 80–85 and the rate for the age group 85+ is higher than that for the age group 80–85. The theoretical curves which approach the horizontal asymptote at or before the age of 80 ($k = 0{\cdot}10$, $p_2/k = 10^{-3}$; $k = 0{\cdot}12$, $p_2/k = 10^{-3}$; $k = 0{\cdot}14$, $p_2/k = 10^{-3}$, $10^{-4}$, $6 \times 10^{-5}$) can be discarded on this ground alone as there is no such approach at the ages under consideration in the observed data. The remaining theoretical curves have been

TABLE II.—*Numerical Values for Expression II taking* $Np_1$ as 10,000 *and Varying* k *and* $p_2/k$.

| $k$ | 0·1 | 0·1 | 0·1 | 0·12 | 0·12 | 0·12 | 0·12 | 0·12 | 0·14 | 0·14 | 0·14 | 0·14 | 0·14 |
|---|---|---|---|---|---|---|---|---|---|---|---|---|---|
| $p_2/k$ | $10^{-3}$ | $10^{-4}$ | $10^{-5}$ | $10^{-3}$ | $10^{-4}$ | $6 \times 10^{-5}$ | $3 \times 10^{-5}$ | $10^{-5}$ | $10^{-3}$ | $10^{-4}$ | $6 \times 10^{-5}$ | $3 \times 10^{-5}$ | $10^{-5}$ |
| $t$ | | | | | | | | | | | | | |
| 20 | 64 | 6·5 | 1 | 100 | 10 | 6 | 3 | 1 | 155 | 15·5 | 6·2 | 3·1 | 1·5 |
| 30 | 200 | 19 | 1·9 | 356 | 35 | 21 | 10 | 3·5 | 657 | 65·7 | 26·2 | 13·1 | 6·6 |
| 40 | 500 | 50 | 5·3 | 1200 | 120 | 72 | 36 | 12 | 2400 | 269 | 107·6 | 53·8 | 26·9 |
| 50 | 1400 | 147 | 15 | 3300 | 400 | 240 | 121 | 40 | 6700 | 1100 | 440 | 220 | 110 |
| 60 | 3300 | 400 | 40 | 7400 | 1300 | 800 | 400 | 100 | 9900 | 3600 | 2200 | 1100 | 360 |
| 70 | 6700 | 1100 | 100 | 9900 | 3600 | 2400 | 1200 | 400 | 10000 | 8400 | 6600 | 4200 | 1600 |
| 80 | 9500 | 2600 | 300 | 10000 | 7700 | 5900 | 3560 | 1400 | 10000 | 10000 | 9900 | 8900 | 5200 |
| 90 | 10000 | 5600 | 800 | 10000 | 9300 | 9100 | 7000 | 4000 | 10000 | 10000 | 10000 | 10000 | 9500 |
| 100 | 10000 | 8900 | 2000 | 10000 | 10000 | 10000 | 9930 | 8700 | 10000 | 10000 | 10000 | 10000 | 10000 |

drawn both in double logarithmic and semi-logarithmic form. The nearest approach to a fit was seen in the family of curves with $k = 0{\cdot}12$. Three of these are shown in Fig. 2 on a semi-logarithmic plot together with the curve from Fig. 1(b) for the observed data. The three theoretical lines are straight up to the age of 70 to 80 when there is a decrease in slope, extension would show a close approach to the horizontal asymptote at the age of 100 to 120. There is a contrast

Fig. 3.—Values for expression II with the parameters $k = 0{\cdot}12$ $p_2/k = 10^{-3}, 10^{-4}, 6 \times 10^{-5}, 3 \times 10^{-5}, 10^{-5}$. Compared with the observed death rate. (Double logarithmic graph.)

with the observed curve which is markedly bowed and shows decreasing slope along its length. Plotting the same curves in double logarithmic form, Fig. 3, shows a contrast between the straight line of the observed line and the curves concave to the left of the theoretical lines.

There is a systematic deviation from a fit between the line drawn from the observed data and those derived from expression II. Not only do the absolute values of the death rates fail to match the theoretical line but there is a difference in the shape of the curves. In a double logarithmic plot the observed line is straight and the theoretical lines are curved. In a semi-logarithmic plot the converse is the case, the observed line is curved and the theoretical lines are, for a large part of their length straight. The occurrence of this systematic deviation is regarded as an important reason for rejecting this mathematical model of the events of carcinogenesis.

Calculation of the absolute values for the constants to be used in expression I shows that the probabilities of each change are reasonable when compared with the probability of genetic changes as the result of the action of known mutagenic agents. A similar calculation can be made for the constants in expression II. The value of $k$, 0·12, is decided by the approximate slope of the observed death rate/age curve. The value of $p_2/k$, approximately $10^{-5}$ is determined by curve fitting; this value gives a value for $p_2$ of $1 \cdot 2 \times 10^{-6}$ which is also within reasonable limits for the probability of a genetic change in a cell. Substituting these values in expression II for the age of 50:

$$120 \times 10^{-6} = Np_1 (40 \times 10^{-4})$$
$$Np_1 = 3 \times 10^{-2}$$

If $N$ is taken as $10^9$ cells the value of $p_1$ becomes $3 \times 10^{-11}$ which is several orders of magnitude below the probabilities determined for expression I and also for the value of $p_2$ calculated above for expression II. If, on the other hand $p_1$ is made equal to $p_2$ at $1 \times 10^{-6}$ the value for $N$, the number of gastric cells at risk becomes $3 \times 10^4$, a number of cells which would occupy only some 3 square millimetres of a single layer of epithelium.

It is suggested on the basis of the systematic lack of fit between the observed data and the calculated curves for expression II and also because of the abnormal probability $p_1$ for the initial genetic change or alternatively the extremely small number of cells to be regarded as susceptible to the first change that the hypothesis expressed mathematically in expression II is untenable and that the multiple hit hypothesis expressed in equation I is to be preferred.

The arguments so far are based on the death rates for England and Wales for the six years 1958 to 1963. Dorn and Cutler (1955) in their monograph on cancer in ten metropolitan areas of the United States gave data on the age incidence of gastric cancer in 1161 women in the year 1947. An abstract of these rates is given in Table III and plots of the age specific incidence against age are given in Fig. 4(a) and (b). Again the double logarithmic plot allows a good fit with a straight line while on the semi-logarithmic plot a straight line cannot be

TABLE III.—*Incidence of Gastric Cancer in Women in* 10 *U.S. Cities* (*from Dorn and Cutler*, 1955)

| Age | No. | Rate/ million | 2 × S.E. |
|---|---|---|---|
| 35— | 14 | 27 | 14·5 |
| 40— | 26 | 55 | 21·6 |
| 45— | 57 | 133 | 35·0 |
| 50— | 82 | 211 | 46·5 |
| 55— | 123 | 372 | 67·0 |
| 60— | 168 | 629 | 97·0 |
| 65— | 206 | 984 | 137·0 |
| 70— | 197 | 1396 | 200·0 |
| 75— | 144 | 1617 | 270·0 |
| 80— | 96 | 2151 | 440·0 |
| 85— | 48 | 2682 | 775·0 |

FIG. 4.—Death rate for gastric cancer in women U.S.A. (from Dorn and Cutler). (a) Double logarithmic graph. (b) Semi-logarithmic graph.

drawn but rather a curve of decreasing slope despite the relatively large standard errors.

The data presented so far represent date contours (Case, 1956) of the mortality experience of women from gastric cancer in Britain and in the U.S.A. It is possible, with a secular trend in the environmental concentrations of carcinogenic agents, that the association shown in Fig. 1 and 3 is artefactual and that the true association should be a straight line in Fig. 2 and 4 and agreement with the mathematical relationship set out in expression II. To elucidate this point the death rates from gastric cancer collected by McKenzie, Case and Pearson (1957; 1965, personal communication) were used in a cohort analysis (Case, 1956). The death rates at different ages for women born in the quinquennia around 1850, 1860, 1870, and 1880 are set out in Table IV. Plots of the age specific death rates

TABLE IV.—*Death Rates per Million for Gastric Cancer in Women by Cohorts (from McKenzie, Case and Pearson,* 1957, 1965)

| | Birth data around | | | |
|---|---|---|---|---|
| Age | 1850 | 1860 | 1870 | 1880 |
| 40— . | | | 098 | 107 |
| 45— . | | 209 | 189 | 169 |
| 50— . | | 331 | 337 | 281 |
| 55— . | 574 | 556 | 507 | 427 |
| 60— . | 818 | 899 | 785 | 634 |
| 65— . | 1293 | 1310 | 1071 | 895 |
| 70— . | 1885 | 1788 | 1575 | 1218 |
| 75— . | 2273 | 1953 | 2006 | |
| 80— . | 2309 | 2371 | 2369 | |
| 85— . | 1804 | 2548 | | |

on double logarithmic and semi-logarithmic graphs (Fig. 5(a) and (b)) show again a straight line in the one and a curve with decreasing slope in the other. The values at the upper extreme of life in the earlier cohorts are lower than would be expected. This may well be due to under-diagnosis in elderly individuals: patients born in 1850 would have been 80 in 1930 when radiological facilities were not so easily available as they are now. These observations confirm the close fit with the mathematical relationship derived in expression (I) and exclude the suggestion that this fit may be artefactual and related to a " cohort effect ".

Fig. 5.—Death rates for gastric cancer in women by cohorts, England and Wales. (a) Double logarithmic graph. (b) Semi-logarithmic graph. The lines on the graph have been separated by shifting along the age axis.

### *An integral number of changes in the " multiple hit " theory*

The plot of the observed values for the death rates correspond closely with those for expression I with a slope which corresponds to an exponent for $t$ of 5·83. The mathematical analysis on which expression I was based predicates a finite number of discrete changes in the cell structure and function before malignancy finally occurs. The number of such changes must be integral and the slope of the age *versus* incidence line on the double logarithmic graph should therefore also be integral. In the case of gastric carcinoma this is not the case, the calculated slope of the best-fitting line is 5·83. A probable explanation of this finding is that one of the series of changes in the cells may occur before birth so that if $n$ is the usual number of changes some individuals, because of their constitution at birth, require only $n - 1$ changes. Expressed algebraically:

IX. $$I = k_2 t^{n-1} + k_1 t^{n-2}$$

which reduces to:

X. $$I = k_2 t^{n-2} \left( t + \frac{k_1}{k_2} \right).$$

If $k_1/k$ is small relative to $t$, $t + k_1/k_2$ will approximate to $t$ and the slope of the line will approximate to that of $t^{n-1}$. If $k_1/k$ is large relative to $t$ $t + k_1/k_2$ will approximate to $k_1/k_2$ and the slope of the line will approximate to that of $t^{n-2}$. In the extreme case, where $k_1/k$ is equal to an intermediate value of $t$ the series of points for expression VI fall between these corresponding to $t^{n-2}$ and $t^{n-1}$ but still fall on a straight line on a double logarithmic plot. Fig. 6 shows such a plot for

FIG. 6.—Lines corresponding to the expressions $I = k_1t^2$, $I = k_2t^2(t + 60)$, $I = k_3t^3$. (Double logarithmic graph.)

the series $I = kt^3 : I = kt^2$ and $I = kt^2\ (t + 60)$ for the values of $t$, 40, 50, 60, 70, 80 which correspond to the ages at which tumours are seen most often in the human. The inference to be drawn is that the most usual number of changes required for malignancy to develop in gastric epithelium is 7 but that some individuals, because of the genetic state of cells of their gastric epithelium at birth, require only 6 changes. Alternatively, if Fisher's modification (Fisher, 1958) is accepted the number of changes required for the development of gastric malignancy in the more usual case would be 4 or 5 and in the exceptional case with an inherited predisposition 3 or 4.

*Development period*

The age at diagnosis or at death is not the age at onset of malignant tumours as there is a clinical development time after the first appearance of a tumour. Allowance for such a period of development will make no difference to the shape of the curves or to the argument in the case of graphs drawn in a semi-logarithmic fashion. If curves are drawn for the age specific death rate observed in England and Wales allowing periods of development of 5 years and 10 years it is seen in Fig. 7 that straight lines of somewhat lesser slope can be drawn to provide a good fit for the points between the ages of 35 years and 80 years. Generally gastric cancer is a neoplasm of rapid development which causes death within a year or two of clinical onset and it is not thought probable that there is prolonged development period in which a fully developed tumour is present in the stomach. This is borne out by the infrequency with which such tumours are incidental findings at necropsy.

FIG. 7.—Death rates for gastric cancer in women. England and Wales 1958–63 assuming development periods of 5 and 10 years. (Double logarithmic graph.)

### DISCUSSION

Analysis of the age specific death rate by the graphical method used here shows a very good and close fit with the curve derived from a multi-stage hypothesis of tumour formation (Fig. 1, 3) (Nordling, 1953; Stocks, 1953; Armitage and Doll, 1954). The fit with the curve derived from expression II is not only quantitatively less good but shows a systematic deviation, instead of being almost a straight line or a curve asymptotic to a straight line at the higher ages there is a systematic difference between the observed and the expected curves. This qualitative difference between the observed and expected curves is present in the cohort analysis, the date contours and the data from the survey of Dorn and Cutler (1955) (Fig. 1(b), 4(b), 5(b)) and indicates that, on mathematical grounds, expression I is to be preferred to expression II as a representation of the observed data on death rate from this cause.

It is now necessary to consider some of the pathological evidence pertinent to distinction between the two stage and multi-stage theories of carcinogenesis. To some extent this distinction is illusory as each of the theories is derived from evidence of different type. The multi-stage hypothesis is based largely on a consideration of data from naturally occurring cancer in the human while the two stage hypothesis is of a qualitatively different nature and is based on the results of animal experiments rather than on clinical data.

The two stages of the two stage hypothesis are, in turn, qualitatively different from each other and may be attributed to the action of carcinogenic substances which may have actions specific only to one stage. These two stages have been designated initiation, the stage in which the cell is altered in such a way that it has a neoplastic potential and promotion, the stage in which, as the result of any of a variety of agents, the potentially malignant cells, which have already undergone initiation, go into a phase of excessive growth (Berenblum and Shubik, 1949). This hypothesis does not exclude the possibility that each of the two stages, in the natural state, may depend on one or several changes in the genotype of the cells.

All cells of the body contain a full set of genetic material in the DNA of the chromosomes and thus have the potential capacity to differentiate in any of the many cell forms seen in the human body both in foetal and in adult life and also have the capacity to multiply at rates far in excess of those seen in the most rapidly growing of malignant neoplasms. In foetal life the rate of growth and of cell multiplication is extremely rapid, if tumours grew at like speed the chance of successful therapy would be much smaller than it is now. The malignant cell is a normal cell which has been changed: it is freed from the control mechanisms which maintain a balance of organ structure; it often has the property of invasion not shared by its normal predecessor; it has, to some extent, autonomy; it is a new type of cell parasitic on the host from which it derived.

We can observe in tumour cells the phenotype which is the expression of the changes which have taken place in the cells both in their genetic material and also in the intra-cellular control mechanisms. Many of these changes are deletions. The function of the tumour cell is usually lost: gastro-intestinal neoplasms often secrete little mucus, lung tumours have no respiratory function, malignant tumours of the endocrine glands often have no hormonal activity. In chronic myeloid leukaemia deletion of part of a chromosome can be seen, the Philadelphia chromosome (Nowell and Hungerford, 1960). More detailed examination has shown a loss of cell surface antigens (Nairn, Richmond, McEntegart and Fothergill, 1960; Tee, Wang and Watkins, 1964). On the other hand the observable changes may be additions to the usual phenotype. Abnormal karyotypes with large numbers of additional chromosomes have been seen in cultured tumour cells (Kirkland, 1966) and tumour specific antigens not present in normal cells have been detected (Nairn *et al.*, 1960; Tee *et al.*, 1964).

Rarely the natural history of a neoplastic disease allows us an opportunity to observe the phenotype of neoplastic tissues at different stages in their life cycle. The pre-cancerous lesions, carcinoma *in situ* of the cervix, Paget's disease of the nipple and Queyrat's erythroplasia of the skin of the penis may be regarded as examples of conditions in which some but not all of the changes of carcinogenesis have occurred. A similar situation may exist in the prostate where I have shown (Ashley, 1965), that the slope of the age specific incidence line of clinical prostatic cancer differs markedly from the slope of the line derived from analysis of the " histological " carcinoma diagnosed by microscopical examination of large numbers of prostates removed at autopsy.

Rather more direct evidence is given by the finding of a series of lesions of increasingly malignant histological appearance in the mucosa of the large intestine. The first of these is the simple polyp clothed by normal rectal mucosa. The second is the so-called adenoma malignum (Lockhart-Mummery and Dukes, 1952) in which some of the cells of the mucosa have lost the function of mucin production and lie as a closely packed, usually darkly staining columnar cell lesion which does not, however, show invasion of the stroma. Thirdly, micro-invasion of the stroma is seen and finally there is gross overt malignancy. Occasionally all stages of this neoplastic progression can be seen in sections from different parts of the same viscus and the suggestion that neoplastic development has been in a stepwise fashion is easy to make.

This sequence of events is not seen in all cases of intestinal carcinoma, most often indeed there is no stage of benign polyp formation. The formation of polyps and the change in the cells of the polyp to adenoma malignum may be compared with the stage of initiation seen in the response of animal tissues to extraneous carcinogens. In hereditary polyposis coli this stage is genetically determined by an abnormal gene which can produce a phenotype otherwise seen as the end result of a series of changes. The alternative form of benign rectal polyp, villous papilloma of the rectum, can develop as the result of a different series of changes and in turn can undergo malignant transformation.

This view that multiple changes in the cell are necessary for the manifestation of a neoplastic potentiality is further supported by the observation of recognizable changes in tumours in the course of their natural history. Occasionally these are clearly shown as differences in karyotype (Ford and Clarke, 1963; Ford, 1963 personal communication) when a number of different lines can be discerned in the tumour cells of cases of reticular neoplasm. More often changes in the phenotype of malignant cells are detectable as differences in histological appearance in different parts of a tumour. In a recent survey of 666 instances of lung cancer in men the histological classification used was simply into well and poorly differentiated squamous cell carcinoma,well and poorly differentiated adenocarcinoma and undifferentiated carcinoma (Ashley and Davies, 1967). In 84 of the 399 tumours with squamous cell differentiation there was a mixture of degrees of differentiation which were often sharply demarcated; similarly among the 73 cases with glandular differentiation 23 showed mixtures of cell types. In 6 instances well and poorly differentiated tumour (5 squamous, 1 adenocarcinoma) as well as undifferentiated tumour was seen. Similar abrupt qualitative changes have also been seen in the cells of a series of primary renal carcinomata and metastatic deposits consisting of only one of two or three types of cell in the primary lesion have been observed.

Such abrupt changes in phenotype in established carcinomata are regarded as analogous to the abrupt changes seen in the epithelium of the rectal mucosa in the series rectal polyp–adenoma malignum–carcinoma which has been outlined above. This stepwise progression by discrete observable changes could be paralleled by similar stepwise changes, not at present detectable in the tumour cell phenotype, during a multi-stage process of carcinogenesis.

The steps of a multi-stage carcinogenesis are often equated to somatic mutations, changes in the genetic material of the cell, and it has been postulated

(Burch, 1962) that the earliest changes may be inherited in nature so that, for example in acute leukaemia in children, there is at birth a section of the population which is liable to develop this neoplasm. His reasoning is based on published family studies and suggests a smaller number of changes (5) in the reticulo-endothelial cells than in the cells of the gastric mucosa. My own calculations show values of $(r - 1)$ close to 6 for chronic lymphatic leukaemia, carcinoma of stomach, oesophagus, pancreas, tongue, skin and bladder and multiple myeloma in the female; values close to 5 for carcinoma of rectum, large intestine, thyroid and vulva; values close to 4 for carcinoma of larynx, small intestine and vagina and tumours of bone, connective tissue and kidney, lymphosarcoma and reticulum cell sarcoma and lower values 2·41 and 2·22 in the case of brain tumours and acute leukaemia in adults.

Some part of the phenotype of a cell is controlled by mechanisms, initially laid down in response to genetic influences from the chromosomes, but subsequently self sustaining. It is conceivable that some of the steps of carcinogenesis may involve changes in these extra nuclear structures and, in response to the action of carcinogens, the probability of change could be substantially higher than the mutation rate in genetic material.

Viral carcinogenesis can be accepted as part of a multi-stage theory of oncogenesis if the viral infection is regarded as capable of inducing one of the stages necessary. It is possible to conceive a situation in which a number of individuals have in their bodies cells which have undergone the first few steps in the progression, when then they are exposed to an oncogenic virus an " epidemic " of tumours could arise.

The multi-stage hypothesis also explains the relatively low incidence of tumours in humans exposed to environmental carcinogens. Not all cigarette smokers develop lung cancer, not all aniline dye workers develop bladder cancer and most of the survivors of Hiroshima did not develop leukaemia. Again individuals in whose cells the earliest changes had occurred before exposure to the carcinogenic substances have a higher risk when exposure occurs.

This concept of multi-stage carcinogenesis unfortunately makes the possibility of there being a " cause " of cancer, in the sense of a necessary cause such as is the case in malaria or tuberculosis, rather remote. This in turn suggests that there are many factors in daily life which are or may be carcinogenic and points out the need for further research in the field of environmental carcinogenesis. The multiplicity of factors concerned also points out the need for research into the several mechanisms by which each of these discrete changes has its effect on the functioning of the cell as a whole.

### SUMMARY

Graphical analysis of the death rates at five year age intervals for gastric cancer in women show a quantitatively and qualitatively better fit with the mathematical expression derived for a multi-stage theory of carcinogenesis than with that derived for a two stage theory. Cohort analysis of the data collected for England and Wales confirms this finding.

The pathological bases for the two theories are considered and it is shown that the two are not mutually exclusive but that the stage of initiation and the stage of promotion may each be the consequence of a number of discrete changes in cell structure and function.

Illustrations are cited to show that after carcinogenesis has occurred further discrete changes in the now neoplastic, autonomous cells can be identified with the comparatively crude methods of light microscopy.

### REFERENCES

ARMITAGE, P. AND DOLL, R.—(1954) *Br. J. Cancer*, **8**, 1.—(1957) *Br. J. Cancer*, **11**, 161.

ASHLEY, D. J. B.—(1965) *J. Path. Bact.*, **90**, 217.

ASHLEY, D. J. B. AND DAVIES, H. D.—(1967) *Cancer, N.Y.*, **20**, 165.

BERENBLUM, I. AND SHUBIK, P.—(1949) *Br. J. Cancer*, **3**, 109.

BURCH, P. R. J.—(1962) *Nature, Lond.*, **195**, 241.

CASE, R. A. M.—(1956) *Br. J. prev. soc. Med.*, **10**, 172.

DORN, H. F. AND CUTLER, S. J.—(1955) *Publ. Hlth. Monogr.* No. 29.

FISHER, J. C.—(1958) *Nature, Lond.*, **181**, 651.

FORD, C. E. AND CLARKE, C. M.—(1963) Canadian Cancer Conference. Vol. 5, p. 129.

KIRKLAND, J. A.—(1966) *Lancet*, i, 152.

LOCKHART-MUMMERY, H. E. AND DUKES, C. E.—(1952) *Lancet*, ii, 751.

McKENZIE, A., CASE, R. A. M. AND PEARSON, J. T.—(1957) General Register Office, Studies on Medical and Population Subjects No. 13. Cancer Statistics for England and Wales 1901–55. London.

NAIRN, R. C., RICHMOND, H. G., McENTEGART, M. G. AND FOTHERGILL, J. E.—(1960) *Br. med. J.*, ii, 1335.

NOWELL, P. C. AND HUNGERFORD, D. A.—(1960) *Science, N.Y.*, **132**, 1497.

NORDLING, C. O.—(1953) *Br. J. Cancer*, **7**, 68.

REGISTRAR GENERAL.—(1960) Statistical Review of England and Wales for the year 1958. Part I. Tables Medical. London (H.M.S.O.).—(1961) Statistical Review of England and Wales for the year 1959. Part I. Tables Medical. London (H.M.S.O.).—(1962) Statistical Review of England and Wales for the year 1960. Part I. Tables Medical. London (H.M.S.O.).—(1963) Statistical Review of England and Wales for the year 1961. Part I. Tables Medical. London (H.M.S.O.).—(1964) Statistical Review of England and Wales for the year 1962. Part I. Tables Medical. London (H.M.S.O.).—(1965) Statistical Review of England and Wales for the year 1963. Part I. Tables Medical. London (H.M.S.O.).

SMITH, C. A. B.—(1961) 'Recent Advances in Human Genetics.' Edited by Penrose, L. S. London (J. and A. Churchill). p. 166.

STOCKS, P.—(1953) *Br. J. Cancer*, **7**, 407.

TEE, D. E. H., WANG, M. AND WATKINS, J.—(1964) *Nature, Lond.*, **204**, 897.